# Biopsy-Confirmed Severe Acute Kidney Injury Requiring Emergent Hemodialysis After Eight Months of Alectinib Therapy

**DOI:** 10.7759/cureus.115121

**Published:** 2026-08-24

**Authors:** Joey H Li, John Jay P Cadavona, Chris Fagel

**Affiliations:** 1 Department of Medicine, David Geffen School of Medicine at UCLA, Los Angeles, USA

**Keywords:** acute kidney injury, alectinib, alk-positive non-small cell lung cancer, anaplastic lymphoma kinase, emergent hemodialysis, tyrosine kinase inhibitor

## Abstract

Anaplastic lymphoma kinase (ALK) inhibitors such as alectinib are standard first-line treatments for ALK-positive lung cancers. While alectinib is generally well tolerated and provides excellent disease control, rare reports have suggested renal toxicities associated with alectinib treatment. Here, we report a case of a 60-year-old man with a history of stage IV non-small cell lung cancer who had been receiving alectinib for eight months and presented to the emergency department with anuria, acutely elevated creatinine, and hyperkalemia. Nephrotoxic medications, including alectinib, were suspended in the setting of severe acute kidney injury (AKI). He was started on emergent hemodialysis and treated with corticosteroids, leading to resolution of the kidney injury and electrolyte abnormalities. Renal biopsy revealed mixed acute tubular necrosis and acute interstitial nephritis. This case reinforces the rare association between alectinib and AKI, emphasizing the importance of monitoring for and recognizing signs of nephrotoxicity in patients receiving this therapy, even after months of stable renal function.

## Introduction

Targeted therapies designed to inhibit driver mutations have dramatically altered the landscape of non-small cell lung cancer (NSCLC) treatment. Mutations in the tyrosine kinase anaplastic lymphoma kinase (ALK) are present in 4-6% of lung adenocarcinomas and drive oncogenic signaling, which can be effectively targeted by ALK inhibitors such as alectinib [1]. Alectinib first received FDA approval for the treatment of metastatic ALK-positive NSCLC refractory to crizotinib in December 2015, followed by approval in the frontline setting in November 2017 [1]. Alectinib demonstrated superior progression-free survival (~34.8 months) compared with its predecessor, crizotinib (10-11 months) [2]. Additionally, alectinib was favored due to its generally mild side-effect profile.

Mild elevations in serum creatinine (Cr) are a recognized and relatively common adverse effect of alectinib, with one retrospective multicenter analysis reporting serum Cr elevation in 7.2% of patients who initiated alectinib [3]. Severe kidney injury associated with alectinib, however, is rare and has typically been reported to present soon after treatment initiation [4,5]. Here, we present a case of acute kidney injury (AKI) requiring emergent hemodialysis (HD) in a patient receiving alectinib for metastatic ALK-positive NSCLC eight months after treatment initiation.

## Case presentation

A 60-year-old man with a history of metastatic NSCLC on a stable dose of alectinib 600 mg twice daily, initiated eight months prior, insulin-dependent type 2 diabetes, hypertension, hyperlipidemia, and gastroesophageal reflux disease presented to the emergency department (ED) with three days of nausea, vomiting, and anuria.

The patient reported a recent cough two weeks ago, for which he was treated empirically for community-acquired pneumonia with intravenous (IV) ceftriaxone in the ED, followed by oral cefpodoxime and azithromycin. Cr was found to be elevated to 1.32 mg/dL at that time from a prior baseline of 0.7 mg/dL. He also reported receiving an infusion of vitamin B12 and vitamin C following this infection. Three days prior to admission, he began to experience nausea, vomiting, severely decreased appetite, and anuria but denied infectious symptoms such as fever, chills, flank pain, dysuria, hematuria, or rash. The medication list was notable for the possible nephrotoxic drugs furosemide and pantoprazole (Table 1).

**Table 1:** Home medications on admission

Medication	Dosage and frequency
Albuterol nebulizer	2.5 mg four times daily as needed
Albuterol inhaler	Two puffs every six hours as needed
Alectinib	600 mg twice daily
Benzonatate	100 mg every eight hours as needed
Betamethasone dipropionate 0.05% ointment	Topical application twice daily
Diphenoxylate-atropine	Two 2.5-0.025 mg tablets four times daily as needed
Docusate	100 mg every night as needed
Furosemide	40 mg every other day
Gabapentin	200 mg every night
Gemfibrozil	600 mg twice daily
Guaifenesin	200 mg three times daily as needed
Insulin glargine	5 units every night
Insulin aspart	3 units daily with breakfast, 5 units daily with lunch
Lisinopril	5 mg daily
Metformin	1000 mg twice daily
Metoprolol tartrate	25 mg twice daily
Multivitamin	1 tablet daily
Pantoprazole	40 mg daily as needed
Pyridoxine	50 mg daily
Sitagliptin	100 mg daily
Vitamin E	400 IU daily

**Figure 1:**
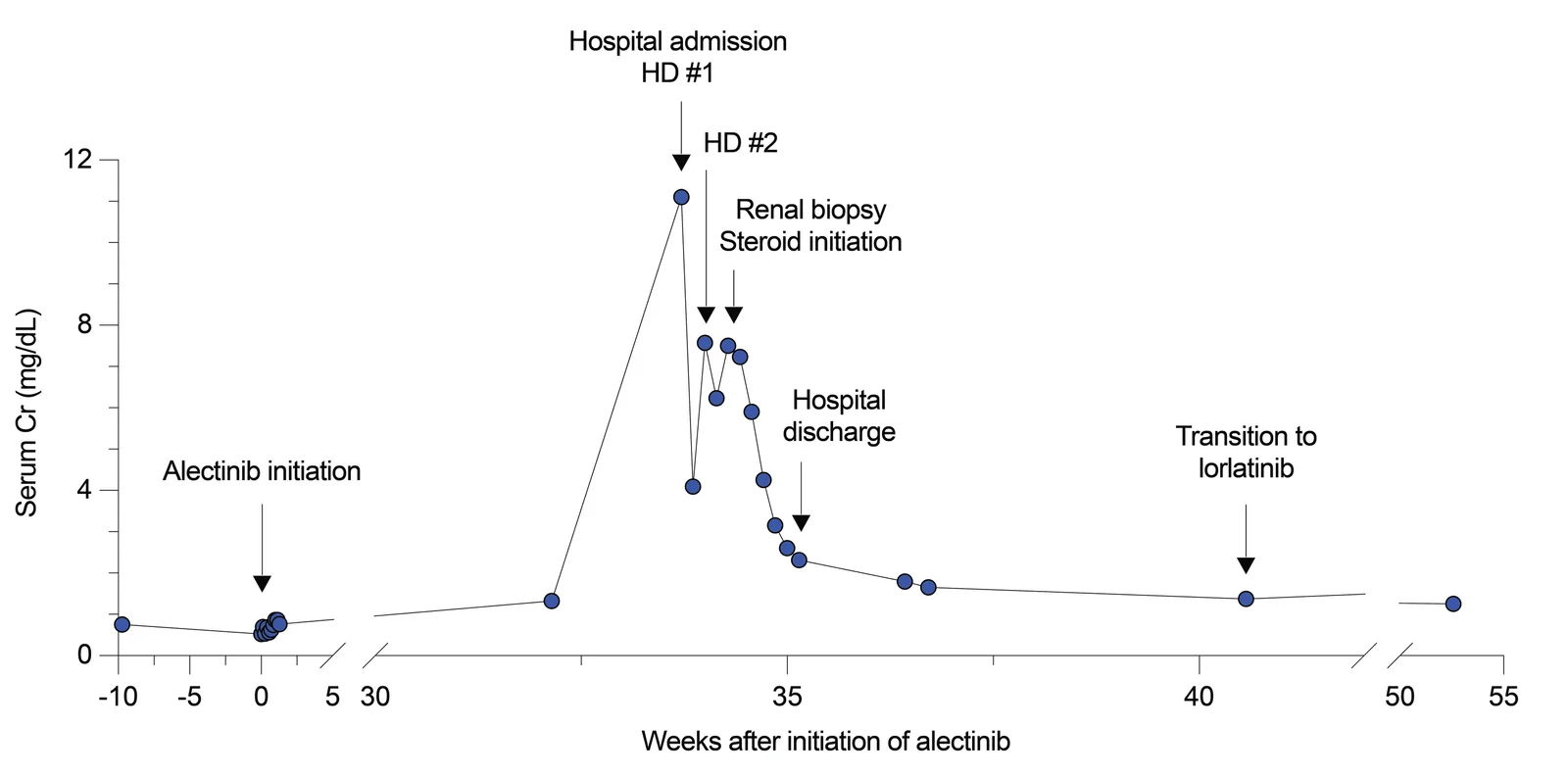
Serum Cr and key clinical events during alectinib treatment and hospitalization Key events highlighted include initiation of alectinib, peak Cr level, hospital admission and HD sessions, renal biopsy, steroid treatment, discharge, and transition to lorlatinib. HD, hemodialysis; Cr, creatinine

On presentation, the patient’s vital signs showed a temperature of 36.6°C, blood pressure of 134/70 mm Hg, heart rate of 79 beats per minute, respiratory rate of 22 breaths per minute, and oxygen saturation of 98% while breathing room air. Physical examination additionally revealed bilateral crackles and bilateral lower extremity edema. A bladder scan confirmed the absence of retained urine. Laboratory studies on admission were significant for severely elevated renal indices, with a Cr of 11.1 mg/dL, blood urea nitrogen (BUN) of 102 mg/dL, potassium of 8.4 mmol/L, and metabolic acidosis with bicarbonate of 14 mmol/L and venous pH of 7.25 (Table 2). As noted, laboratory studies obtained 12 days prior demonstrated a Cr of 1.32 mg/dL and a normal serum potassium level (Figure 1). An electrocardiogram was performed to evaluate for cardiac complications of the patient’s electrolyte abnormalities and showed normal sinus rhythm.

**Table 2:** Basic metabolic panel on admission and after hemodialysis Creatinine kinase was not measured after hemodialysis. GFR, glomerular filtration rate

Blood work test	Test result on initial presentation	Test result after hemodialysis	Reference range
Sodium	132 mmol/L	137 mmol/L	135-146 mmol/L
Potassium	8.4 mmol/L	4.1 mmol/L	3.6-5.3 mmol/L
Chloride	97 mmol/L	98 mmol/L	96-106 mmol/L
Total CO_2_	14 mmol/L	23 mmol/L	20-30 mmol/L
Anion gap	21 mmol/L	16 mmol/L	8-19 mmol/L
Urea nitrogen	102 mg/dL	45 mg/dL	7-22 mg/dL
Creatinine	11.10 mg/dL	5.91 mg/dL	0.60-1.30 mg/dL
Estimated GFR	5 ml/min/1.73 m^2^	10 ml/min/1.73 m^2^	60-89 ml/min/1.73 m^2^
Glucose	120 mg/dL	137 mg/dL	65-99 mg/dL
Creatinine kinase	220 U/L		63-473 U/L

**Figure 2:**
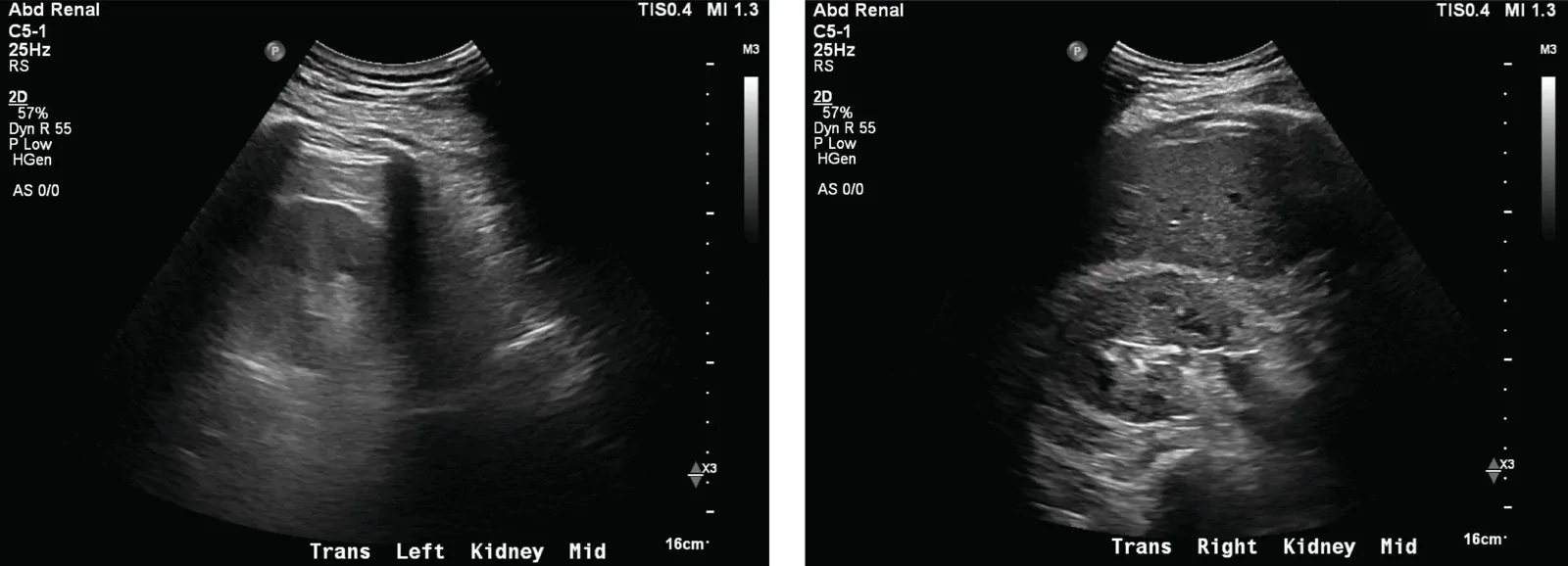
Renal ultrasound images obtained on admission Transverse views of the left and right mid kidneys through the renal pelvis. Ultrasound revealed mild bilateral hyperechogenicity with no hydronephrosis.

These overall findings met criteria for Kidney Disease: Improving Global Outcomes (KDIGO) stage 3 AKI requiring renal replacement therapy. The initial broad differential diagnosis for the patient’s severe AKI included volume depletion in the setting of recent infection and poor oral intake, infection, rhabdomyolysis, postrenal obstruction, and medication toxicity. Urine cultures were collected and were negative for bacterial growth. Renal ultrasound showed no obstruction or hydronephrosis but was notable for mildly hyperechoic kidneys suggestive of parenchymal disease (Figure 2). Cr kinase levels were normal (Table 2). The patient’s medications were reviewed for possible nephrotoxins, and furosemide, pantoprazole, lisinopril, and alectinib were held on admission given their possible involvement in the patient's renal injury. Initial temporization of hyperkalemia was achieved by administration of insulin, albuterol, IV dextrose, and normal saline, and a temporary HD catheter was placed. Nephrology was consulted, and the patient was admitted to the intensive care unit for emergent HD to definitively correct his hyperkalemia. Following an initial two-hour session with 2 L of ultrafiltrate removed, potassium normalized to 4.1 mmol/L and bicarbonate improved to 23 mmol/L, with partial improvement in renal function (Cr 5.91 mg/dL, BUN 45 mg/dL) (Table 2). The patient was able to produce a small amount of urine for urinalysis, which revealed 1+ blood, 2+ glucose, 1+ protein, a urine albumin/Cr ratio of 2282.5, a urine protein/Cr ratio of 5.2, and negative eosinophils (Table 3). Despite this, the patient remained persistently oliguric with clinical evidence of volume overload and worsening renal indices on the following day, with Cr rising to 7.57 mg/dL and BUN to 53 mg/dL. A subsequent one-hour HD session was performed with removal of 1 L of ultrafiltrate.

**Table 3:** Urinalysis on admission and after hemodialysis Albumin and albumin/creatinine ratio were not measured on urinalysis post-hemodialysis. Bili, bilirubin; RBC, red blood cells; WBC, white blood cells; HPF, high-powered field; LPF, low-powered field

Urine test	Test result on initial presentation	Test result after hemodialysis	Reference range
Urine color	Yellow	Colorless	None specified
pH	5.5	7.5	5.0-8.0
Specific gravity	1.012	1.005	1.005-1.030
Blood, dipstick	1+	1+	Negative
Bili, dipstick	Negative	Negative	Negative
Glucose	2+	2+	Negative
Ketones	Negative	Negative	Negative
Protein	1+	1+	Negative
Nitrite	Negative	Negative	Negative
Leukocyte esterase	1+	Negative	Negative
RBC per uL	15 cells/μL	1 cell/μL	0-11 cells/μL
WBC per uL	54 cells/μL	5 cells/μL	0-22 cells/μL
Eosinophils	Negative	Negative	Negative
RBC per HPF	3 cells/HPF	0 cells/HPF	0-2 cells/HPF
WBC per HPF	10 cells/HPF	1 cells/HPF	0-4 cells/HPF
Squamous epithelial cells	4 cells/μL	0 cells/μL	0-17 cells/μL
Hyaline casts	0-2/LPF	0-2/LPF	0-2/LPF
Creatinine	38.9 mg/dL	19.9 mg/dL	None specified
Albumin	887.9 mg/L		None specified
Albumin/creatinine ratio	2282.5 mcg/mg		<30.0 mcg/mg
Protein	194 mg/dL	37 mg/dL	None specified
Protein/creatinine ratio	5.2 g/g	1.9 g/g	0-0.4 g/g

**Figure 3:**
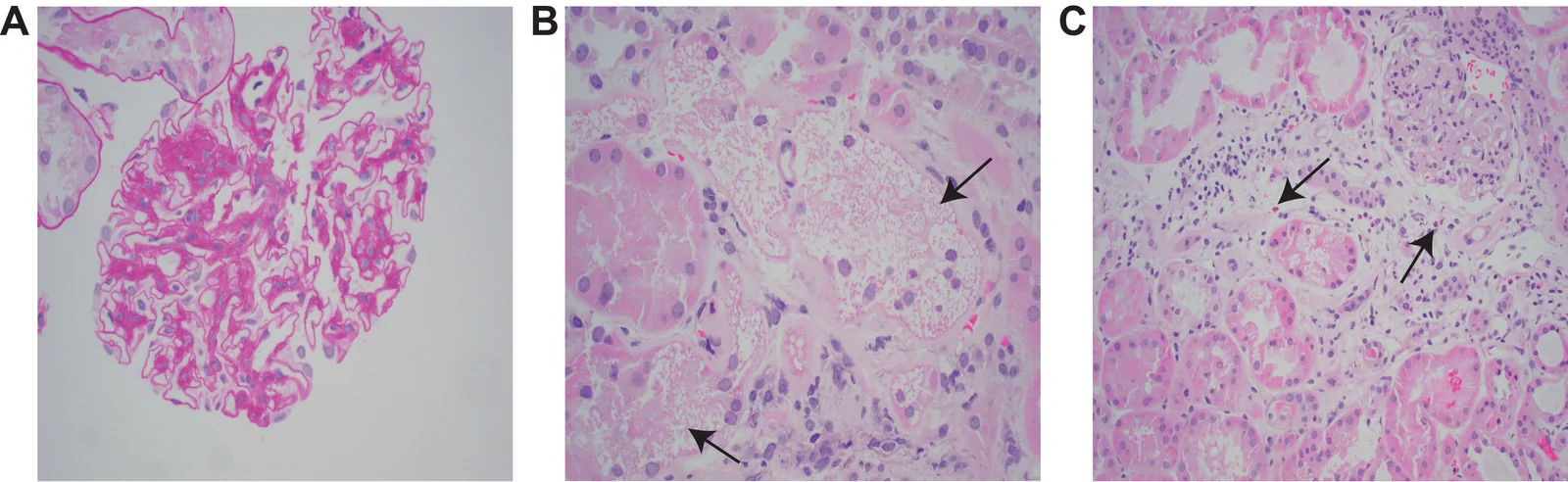
Representative microscopy images of renal biopsy histopathologic findings Renal tissue staining with (A) periodic acid-Schiff stain showing early nodular mesangial sclerosis; (B) hematoxylin and eosin stain showing tubular cytoplasmic vacuolization, with arrows indicating areas of vacuolization; and (C) hematoxylin and eosin stain showing mild tubulointerstitial inflammation with eosinophils, with arrows highlighting infiltrating eosinophils. Magnification: 400×.

Due to the patient’s persistently worsening renal function, oncology was consulted regarding potential alectinib-associated nephrotoxicity. A renal biopsy was subsequently performed. Preliminary pathology suggested acute interstitial nephritis (AIN), and systemic corticosteroid therapy with oral prednisone was initiated at a dose of 1 mg/kg daily starting the day after the biopsy. Final histopathologic evaluation demonstrated acute tubular necrosis (ATN) with prominent cytoplasmic vacuolization, accompanied by mild acute tubulointerstitial nephritis with eosinophilic infiltration (Figure 3). Additional findings included moderate diabetic glomerulopathy with early nodular formation, focal global glomerulosclerosis (~32%), and mild interstitial fibrosis/tubular atrophy (20-25%), despite the patient having no prior history of diabetic nephropathy. No immune complex-mediated process was identified. Following initiation of corticosteroid therapy, the patient’s Cr peaked at 7.50 mg/dL before progressively improving. Repeat urinalysis showed improvement in proteinuria (Table 3). HD was discontinued, and the dialysis catheter was removed six days after initial placement.

At discharge, Cr improved to 2.32 mg/dL, and urine output improved to 0.7 mL/kg/hr (Figure 1, Table 4). The patient continued prednisone 1 mg/kg for three weeks as an outpatient, after which steroids were tapered by 10 mg every five days until reaching a dose of 10 mg, then continued at 5 mg for five days and 2.5 mg for five days before discontinuation. The patient also continued trimethoprim/sulfamethoxazole for Pneumocystis pneumonia prophylaxis until completion of the steroid taper. Renal function subsequently showed partial recovery, with Cr between 1.2 and 1.3 mg/dL at follow-up six weeks after discharge (Figure 1). As an outpatient, the patient was switched to lorlatinib therapy and has since maintained stable renal function at this new baseline.

**Table 4:** Urine output during admission

Day of hospitalization	24 hour total urine output (mL)	24 hour urine output rate (mL/kg/hr)
1	60	0.0
2	250	0.1
3	1200	0.6
4	750	0.4
5	1475	0.8
6	1725	0.9
7	925	0.5
8	2750	1.5
9	1200	0.7

## Discussion

ALK gene rearrangements are present in approximately 4% of patients with NSCLC [6]. The first-generation ALK inhibitor crizotinib was initially established as first-line therapy for ALK-positive NSCLC based on superior progression-free survival and response rates compared with chemotherapy in the PROFILE 1014 trial [7]. Second-generation ALK inhibitors such as alectinib were subsequently developed to provide enhanced central nervous system (CNS) penetration and demonstrated improved outcomes and control of CNS disease progression compared with crizotinib in multiple clinical trials [8-10]. Alectinib also proved superior to standard-of-care chemotherapy in the adjuvant setting, with 93.8% of patients receiving alectinib remaining disease-free at two years compared with 63% of chemotherapy-treated patients [11]. Newer inhibitors such as lorlatinib have been further refined to overcome resistance mechanisms observed with first- and second-generation ALK inhibitors [12,13].

In the ALEX study, patients treated with alectinib developed grade 3-5 toxicities in 41% of cases [10]. These adverse events primarily included transient transaminitis, hyperbilirubinemia, and anemia. Only four of 152 patients (2.6%) receiving alectinib in the ALEX trial experienced renal toxicities [14]. In the J-ALEX trial, 11% of patients receiving alectinib had increased Cr levels, but none experienced grade 3-4 toxicities [8]. Severe renal toxicities have rarely been reported in the literature. Ramachandran et al. described the onset of acute renal failure in a patient six weeks after initiation of alectinib 600 mg twice daily for ALK-positive lung adenocarcinoma, which required HD. Renal failure in this case was thought to be due to ATN, although no biopsy was obtained [4]. This case was notable in that the patient was re-challenged with alectinib upon recovery of renal function, leading to another acute increase in Cr and strongly suggesting that alectinib was the precipitating renal insult. Another report describes a similar case of AKI 27 days after starting alectinib, which resolved after treatment with HD and corticosteroids [5]. Renal biopsy demonstrated mixed AIN and acute tubular injury, a pattern similar to the biopsy findings in our case. This patient was also transitioned to lorlatinib and subsequently had continued stable renal function.

In contrast to these published cases, our patient was on alectinib for eight months with stable Cr before developing worsening renal function, suggesting that the kinetics of alectinib-induced renal toxicity vary widely among patients. Renal biopsy revealed mixed AIN and ATN despite negative eosinophils on urinalysis, suggesting the low sensitivity of urine eosinophils for AIN, as well as histopathologic evidence of underlying chronic diabetic glomerulonephropathy. Indeed, we must highlight that the patient presented with multiple additional renal risk factors, such as underlying diabetic nephropathy, recent respiratory tract infection, and concomitant nephrotoxic medications, producing a multifactorial clinical picture that predisposed the patient to renal failure. Despite these risk factors, evaluation of this case using the Naranjo Adverse Drug Reaction Probability Scale suggested that alectinib was the probable cause of the patient’s AKI (Table 5) [15]. Urgent HD together with corticosteroids and supportive care was sufficient to achieve dialysis independence and partial renal recovery. Together, these cases strongly indicate that alectinib can precipitate severe, reversible kidney injury in select patients. Early stability of renal function may not rule out delayed onset of renal toxicity, and obtaining baseline Cr measurements and routine monitoring of renal function, particularly in the setting of chronic renal risk factors such as diabetes or acute precipitants such as volume depletion, may be warranted in patients receiving alectinib. Clinicians should maintain a high suspicion for alectinib-induced renal toxicity in patients receiving alectinib who present with AKI, and these patients may benefit from transitioning to an alternative ALK inhibitor.

**Table 5:** Naranjo scale for adverse drug reaction probability Probability was scored according to the Naranjo Adverse Drug Reaction Probability Scale. Total score was interpreted based on the following: ≤0: doubtful; 1-4: possible; 5-8: probable; ≥9: definite. For question 4, a response of “yes” applied due to prior published recurrence of AKI upon alectinib rechallenge, providing a clinical contraindication to resuming alectinib in this patient.

Question	Response	Score
Are there previous conclusive reports on this reaction?	Yes	+1
Did the adverse event appear after the suspected drug was administered?	Yes	+2
Did the adverse event improve when the drug was discontinued, or a specific antagonist was administered?	Yes	+1
Did the adverse event reappear when the drug was readministered?	Yes	+2
Are there alternative causes that could on their own have caused the reaction?	Yes	-1
Did the reaction reappear when a placebo was given?	Do not know	0
Was the drug detected in blood or other fluids in concentrations known to be toxic?	Do not know	0
Was the reaction more severe when the dose was increased or less severe when the dose was decreased?	Do not know	0
Did the patient have a similar reaction to the same or similar drugs in any previous exposure?	Do not know	0
Was the adverse event confirmed by any objective evidence?	Yes	+1
Total score		6

The mechanism of alectinib-induced renal toxicity remains poorly understood. Renal toxicity and ATN associated with crizotinib have similarly been reported, suggesting a possible class-wide effect [16]. Interestingly, alectinib is primarily metabolized by cytochrome P450 (CYP450) 3A4 in the liver, and isotope-labeled alectinib administered to human subjects was almost entirely excreted in the feces rather than the urine [17,18]. Biopsy results from our patient revealed tubular vacuolization, which classically suggests a mechanism of osmotic tubular damage [19]. Indeed, a similar histologic presentation has been observed in prior reports of alectinib-associated renal failure [20,21]. As reports of ALK inhibitor-induced kidney injury remain rare, patient-specific factors such as comorbid medical conditions or underlying genetic polymorphisms may also contribute to alectinib toxicity. Preclinical studies in mice have shown that mutations in CYP450 reductase led to worsened renal function with renal tubular vacuolization after exposure to nephrotoxic chemotherapeutics, suggesting that individuals with such gene variants may be more sensitive to alectinib-induced renal injury [22]. Further study will be needed to determine the cellular mechanism underlying nephrotoxicity associated with alectinib and other ALK inhibitors.

## Conclusions

Alectinib-associated nephrotoxicity is rare but may present as severe, reversible AKI requiring renal replacement therapy despite months of previously stable treatment. This case demonstrates that delayed-onset renal injury can occur despite an initially stable Cr trajectory. Clinicians should routinely monitor renal function in patients receiving alectinib therapy, particularly those with additional risk factors for kidney injury, such as underlying diabetic kidney disease, as earlier identification may prevent progression to life-threatening renal failure and provide an opportunity to change therapy to an alternative ALK inhibitor such as lorlatinib. In cases of suspected alectinib-related kidney injury, prompt drug discontinuation and supportive management, including HD and corticosteroids, may lead to substantial recovery.
